# The Potential-Dependent Structure of Pt_3_Ni Alloy Electrocatalysts and Its Effect on Electrocatalytic Activity

**DOI:** 10.1021/acscatal.5c02601

**Published:** 2025-07-16

**Authors:** Hassan Javed, Kees Kolmeijer, Nipon Deka, Matthijs A. van Spronsen, Marijn A. van Huis, Athira Lekshmi Mohandas Sandhya, Ivan Khalakhan, Rik V. Mom

**Affiliations:** † Leiden Institute of Chemistry, 4496 Leiden University, PO Box 9502, Leiden 2300 RA, The Netherlands; ‡ Diamond Light Source Ltd., Harwell Science and Innovation Campus, Chilton, Didcot OX11 0DE, U.K.; § Debye Institute for Nanomaterials Science, Utrecht University, Princetonplein 5, 3584 CC Utrecht, The Netherlands; ∥ Department of Surface and Plasma Science, Faculty of Mathematics and Physics, 37740 Charles University, V Holešovičkách 2, 180 00 Prague 8, Czech Republic

**Keywords:** platinum−nickel electrocatalyst, alloy electrocatalyst, X-ray photoelectron spectroscopy, X-ray absorption spectroscopy, spectro-electrochemistry

## Abstract

The distribution of elements within alloy nanoparticles is a critical parameter for their electrocatalytic performance. Here, we use the case of a Pt_3_Ni alloy to show that this elemental distribution can dynamically respond to the applied potential, leading to strongly potential-dependent catalytic properties. Starting from the Pt_3_Ni core and Pt shell structure that forms in acid electrolyte due to Ni leaching, our electrochemical X-ray photoelectron spectroscopy measurements show that the Ni atoms can be reversibly moved between the core of the particles and the near-surface region using the applied potential. Through potential jump measurements, we show that this Ni migration modulates the hydrogen evolution reaction activity of the particles by over 30%. These observations highlight the potential of incorporating in situ restructuring of alloys as the final step in electrocatalyst design.

## Graphical abstract

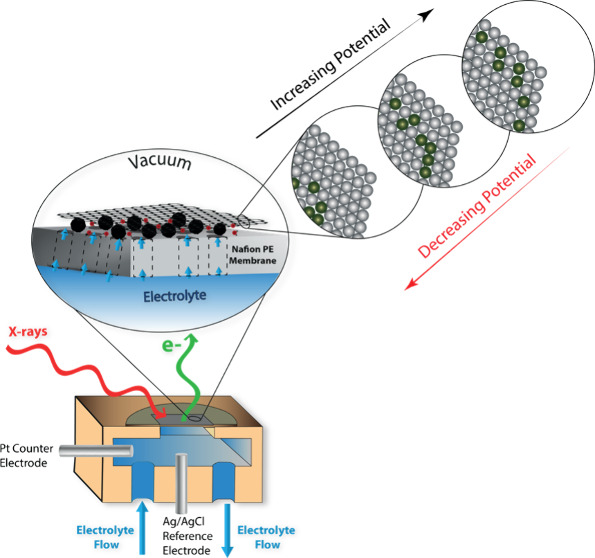

## Introduction

Platinum-based alloys such as Pt_3_Ni are a core component of modern fuel cells, serving as the oxygen reduction reaction (ORR) catalyst on the cathode side.^1–4^ Compared to pure Pt electrocatalysts used in older generations, these alloys show significantly better performance.^5–12^ Similar improvements have been observed for other reactions, such as methanol oxidation^13,14^ and hydrolytic hydrogenation.^15^ The origin of these improvements can be attributed to strain and ligand effects in the alloys, which modulate the electronic structure of the active Pt surface atoms.^16^ Briefly, the ligand effect^17,18^ refers to the change in the electronic structure of the Pt atoms due to the proximity of the alloying metal atoms, whereas the strain effect points to the reduction of the interatomic distance of the Pt metal atoms due to the introduction of relatively smaller transition metal (e.g., Pt: 0.139 nm, Ni:0.124 nm^19^) in the matrix, causing the compression of the surface atomic structure. Theoretical studies^20–23^ have shown that using the strain and ligand effects, the d-band center can be shifted to a more favorable position in alloys with respect to pure Pt. This optimizes the binding strength of the reaction intermediates on the surface and thus enhances the activity.^20–22^

To exploit the strain and ligand effects in electrocatalyst design, it is critical to consider the evolution of the Pt-alloy structure under electrochemical conditions. For example, for Pt–Ni alloys in acidic electrolyte, the Ni atoms leach out at the surface, leading to the formation of a pure Pt shell and a Pt-alloy core.^23–25^ An inverse correlation between the ligand and strain effects and the Pt shell thickness is observed in the literature,^26^ with the compressive strain exerted on the surface Pt atoms as well as the electronic structure modification due to the ligand effect scaling down with thicker Pt shells. Therefore, any enhancement due to ligand and strain effects, such as higher ORR activity, also depends inversely on the thickness of this Pt shell,^16,17^ making the shell formation properties of Pt-alloy electrocatalysts a critical design parameter.^26,27^

Here, we hypothesized that the nature of the Pt shell in electrochemical systems may depend not only on the electrolyte pH but also on the applied potential. This hypothesis is based on the alloy restructuring observed in heterogeneous catalysis, where elemental redistribution within the catalyst particles occurs, depending on the adsorbates delivered from the gas phase. For example, in Pd–Rh alloys, it was shown that Rh is drawn to the surface in an oxidizing NO environment, whereas a Pd–Rh mixture is formed at the surface in a CO atmosphere.^28^ Importantly, these effects are reversible, meaning that the particle dynamically equilibrates to its environment. Similar restructuring has been observed for Pt bimetallic catalysts in gas-phase systems when the catalyst is thermally oxidized (drawing out, e.g., Ni) or reduced (bringing Pt to the surface).^23,29–31^ Here, we studied whether such reversible restructuring can also occur under electrochemical conditions, where the surface adsorbate structure is strongly dependent on the applied potential.

To observe the elemental distribution within Pt-alloy nanoparticles under electrochemical conditions, we employed an electrochemical X-ray photoelectron spectroscopy (XPS) approach based on a graphene-covered membrane electrode assembly. Taking the example of a Pt_3_Ni alloy, we demonstrate that its structure is highly dynamic under varying applied potentials, resulting in a potential-dependent catalytic activity.

## Methods

### In Situ X-ray Spectroscopy

To conduct the electrochemical XPS measurements, a graphene-covered membrane electrode assembly (MEA) (Figure 1a) was used in an in-house designed spectro-electrochemical cell. The detailed construction of the cell, as well as the MEA, has been discussed in our earlier work^32–34^ and is described in detail in the Supporting Information (SI Sections 3 and 6). Briefly, the MEA is made up of ∼5 nm Pt_3_Ni catalyst nanoparticles sputter-deposited onto a cleaned and activated Nafion membrane and then coated with a bilayer graphene window. This graphene window is transparent for the incoming X-rays and escaping photoelectrons, permitting XPS measurements, and provides electrical contact with the catalyst. During the in situ measurements, the membrane is wetted from the back by the electrolyte (Ar-purged 0.1 M sulfuric acid, Sigma-Aldrich 99.9% purity). This electrolyte permeates through the proton-exchange membrane (Nafion) to the catalyst. The key function of the graphene window is to impede the evaporation of this electrolyte into the vacuum of the XPS chamber, so that XPS measurements can be conducted under wet electrochemical conditions.

**Figure 1:**
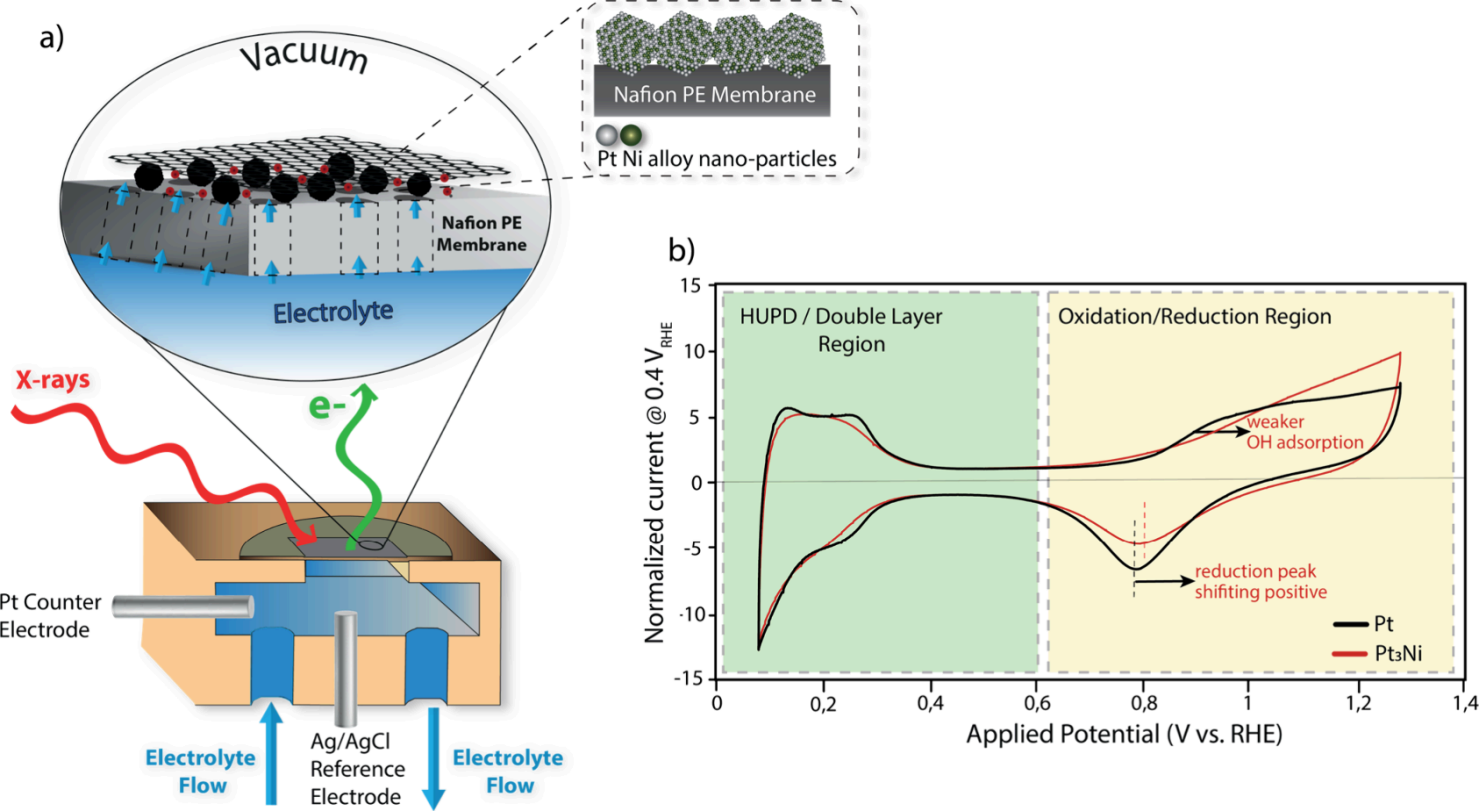
(a) Cell for electrochemical XPS measurements containing an MEA consisting of the Pt_3_Ni catalyst sandwiched between a Nafion membrane and a bilayer graphene. (b) Cyclic voltammograms of Pt and Pt_3_Ni catalysts recorded in the XPS cell in 0.1 M H_2_SO_4_ at 50 mV s^–1^, showing hydrogen underpotential deposition (HUPD), double layer (DL), and oxidation/reduction regions.

To conduct the measurements, the near ambient pressure end station B-07C at Diamond Light Source, UK, was utilized.^35^ The XPS signal was collected using the SPECS Phoibos NAP analyzer, whereas the total electron yield X-ray absorption spectroscopy (XAS) at the Ni L-edge was conducted via the current collected on the analyzer cone. Care was taken to avoid beam damage on the Nafion membranes. Therefore, each spectrum was recorded at a new location on the MEA.

The data for XPS were processed using the CASA XPS software, and Athena was used for XAS. Details of the XPS and XAS data analyses are presented in the Supporting Information (SI).

### Electron Microscopy

The pristine samples were analyzed using electron microscopy, using a TFS Spectra 300 TEM, equipped with monochromator and double aberration correction, and operated at 300 kV in STEM (scanning transmission electron microscopy) imaging mode with a semiconvergence angle of 20.2°. For STEM-HAADF (high-angle annular dark field) imaging, a probe current of 0.2 nA and a dwell time of 10 μs were used. Chemical mapping was performed using STEM-EDS (energy-dispersive X-ray spectroscopy) measurements, where a probe current of 1.6 nA and a dwell time of 2.5 μs were used while the region of interest was scanned continuously during 6–8 min to acquire the Pt, Ni compositional maps.

### Electrochemical Measurements

An Ag/AgCl reference electrode (eDAQ Leakless Miniature Ag/AgCl Reference Electrode), Pt counter electrode, and BioLogic potentiostat (SP-200) were used in the XPS setup. For reporting purposes, all of the potentials mentioned in this work are converted to the RHE scale. Prior to the measurement, the potential was cycled between 0.1 V_RHE_ and 1.2 V_RHE_ at 50 mV s^–1^ to prevent any memory effects and to aid the leaching of the unstable Ni atoms from the surface layer of the bimetallic catalyst particles.^36^ For the chronoamperometry series, the potential was stepped between 0.1 and 1.3 V_RHE_.

Supporting glass cell measurements were conducted with a similar arrangement. However, the catalyst was deposited onto a polished glassy carbon disc (preparation described in SI-2) and measurements were conducted using a reversible hydrogen electrode (RHE) as a reference. All the measurements were done in a hanging meniscus configuration, and the electrochemical data was *iR* corrected at 85%.

## Results and Discussion

The structure and elemental distribution of the pristine Pt_3_Ni samples was analyzed using transmission electron microscopy. The STEM-HAADF images displayed in Figure 2a–d and Figure S5 show that the Pt_3_Ni particles are distributed as a uniform and continuous single-layer nanoparticle film on the Nafion, where the nanoparticles have a size range of about 3–4 nm. Energy-dispersive X-ray spectroscopy (STEM-EDS) was also performed on the pristine samples to verify that Pt and Ni are alloyed and evenly distributed, as shown by the Pt and Ni chemical maps and their overlay images (Figure 2 b-d)

**Figure 2:**
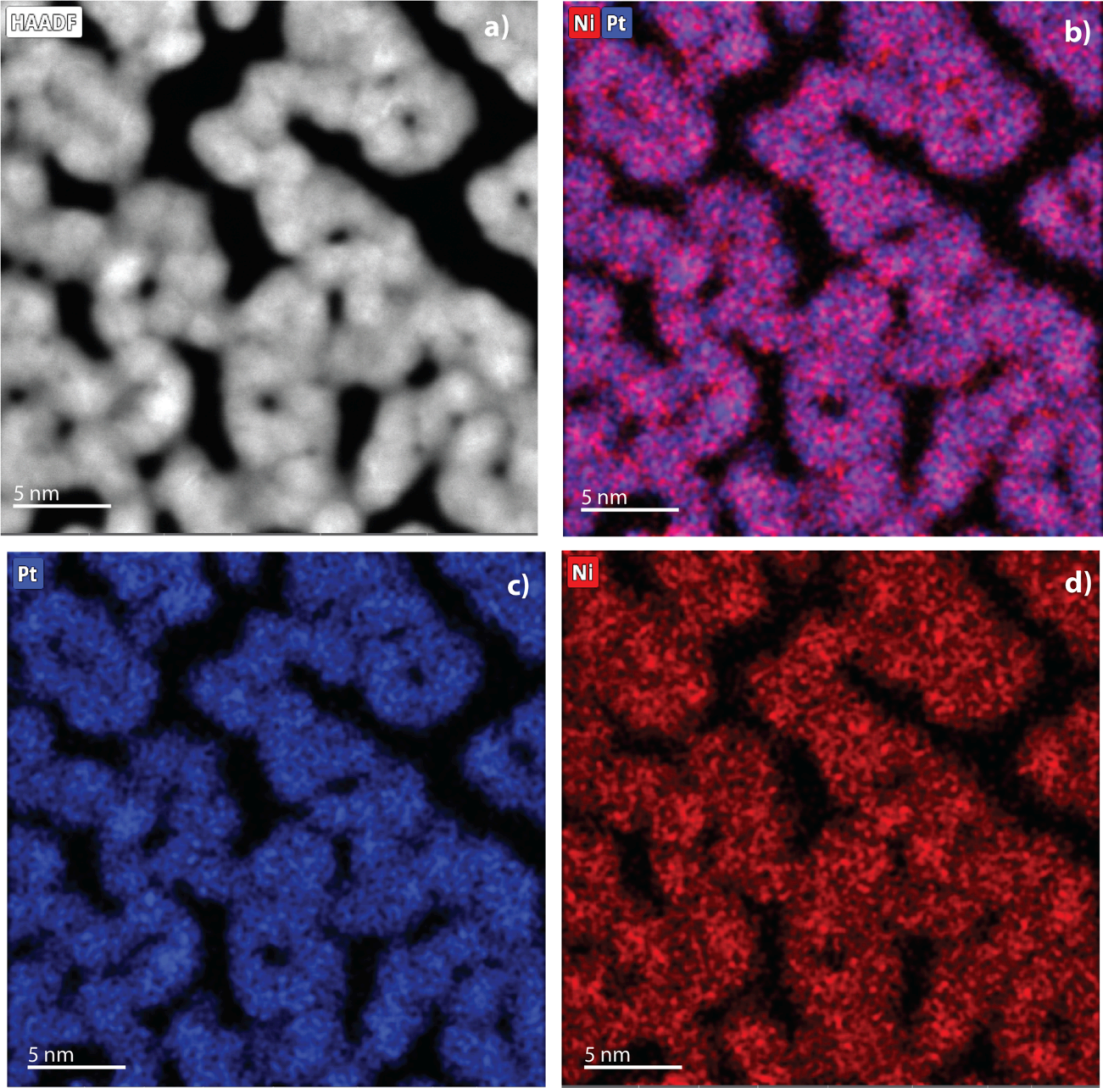
(a) High-resolution TEM image of Pt3Ni nanoparticles supported on Nafion-coated Cu TEM grid. (b–d) STEM-EDS images showing elemental maps of Pt+Ni, Pt, and Ni, respectively, indicating an even distribution of Pt and Ni across the sputtered surface.

We then studied the electrochemical behavior of our Pt_3_Ni catalyst. Figure 1b shows cyclic voltammograms (CVs) of Pt and Pt_3_Ni particles recorded in the spectro-electrochemical cell (complementary experiments in the glass cell are shown in SI Section 11). The current for both curves is normalized to the double-layer current at 0.4 V_RHE_ for the sake of comparison. In the oxidation region, it can be seen that the onset of oxidation, attributed to OH adsorption, is delayed for the Pt_3_Ni particles compared to pure Pt, in line with the literature.^7,37^ This indicates weakened adsorbate bonding, which is considered favorable for ORR because strongly bonded adsorbates function as catalyst poison in the reaction.^40,41^ Other CV features frequently observed in the literature for Pt–Ni alloys, such as the PtO_*x*_ reduction peak shifting slightly positive,^38,39^ are also seen. Overall, these differences between Pt and Pt_3_Ni highlight that Ni has a clear impact on the surface chemistry, in line with the literature.^42,43^

As a next step, we studied the structures of MEA and Pt_3_Ni particles using electrochemical XPS. Figure 3a shows a survey XPS spectrum recorded at the open circuit potential (OCP, 760 mV vs RHE) at *h*ν = 1400 eV. The expected contributions from the Nafion polymer electrolyte membrane (S 2*s*/2p, C 1s, O 1s, and F 1s), the catalyst (Pt 4f), and the graphene window (C 1s) can be prominently seen. Notably, no Ni 2p contribution was observed at ∼852–854 eV, indicative of the Ni leaching from the surface of the particles that is expected in acidic electrolyte.^40–44^ Such leaching leads to the formation of a core–shell structure, with a Pt shell and an alloy core.^45^ Indeed, Ni 2p spectra recorded at 1400 and 1900 eV (Figure 3b) show that for a higher probing depth (*h*ν = 1900 eV), Ni is more clearly visible, consistent with a core–shell structure where the Ni resides in the core. Based on the intensity ratio of the two Ni 2p spectra and the electron attenuation lengths calculated for Pt,^46^ the Pt shell thickness is estimated to be roughly 1 nm (Figure 3c). While this leached core–shell structure will contain less Ni than the initial Pt_3_Ni composition, we refer to the particles as Pt_3_Ni particles for simplicity.

**Figure 3:**
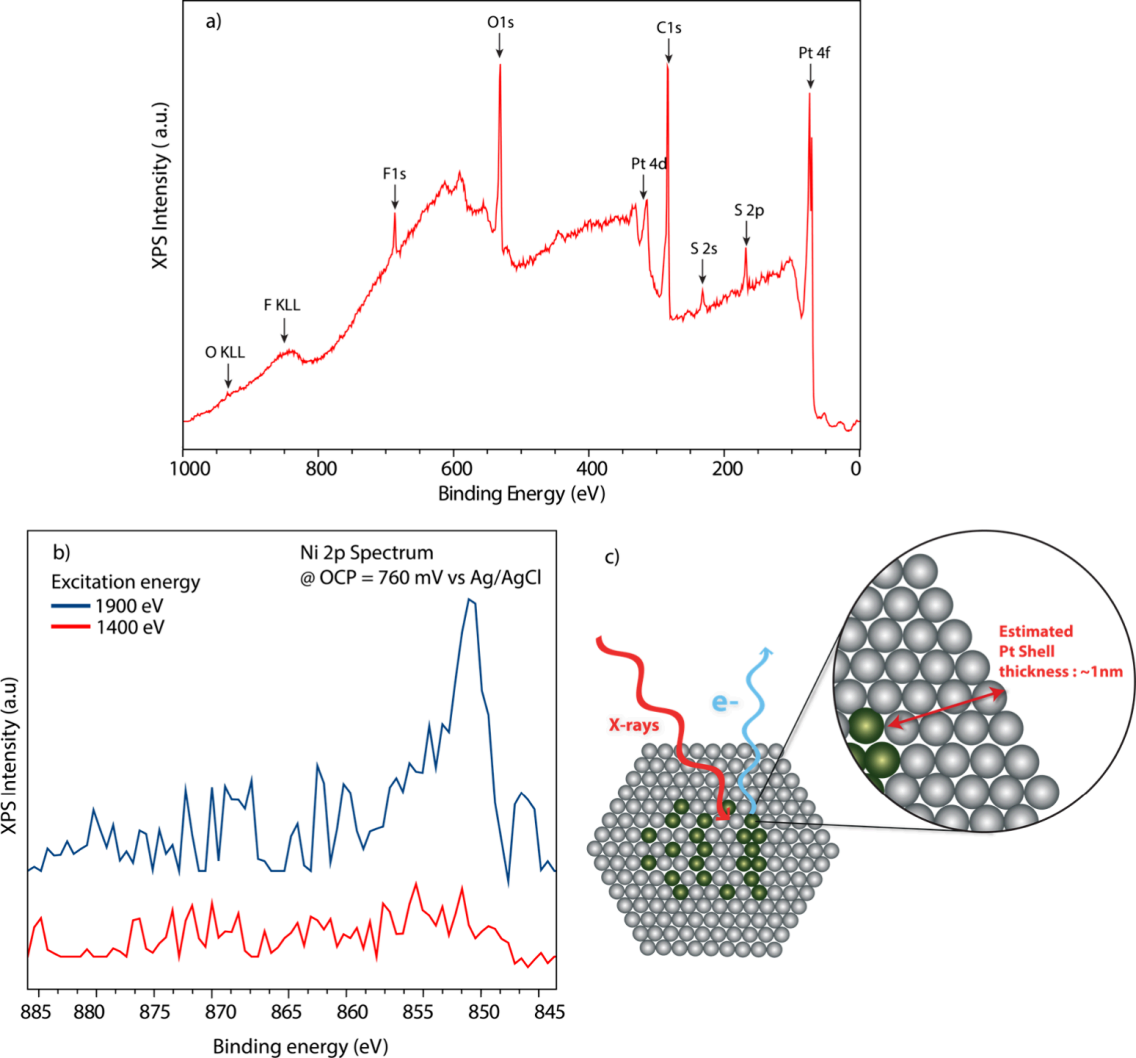
(a) Survey spectrum of OCP Pt_3_Ni MEA highlighting the contributions from the membrane (S 2p, F 1s, O 1s, and C 1s), graphene overlayer (C 1s), and the catalyst (Pt 4f), measured at 1400 eV. (b) Ni 2p showing a noticeable nickel contribution at a higher X-ray excitation energy (1900 eV) while almost no signal is seen at lower excitation energy (1400 eV). (c) Schematic impression of the in situ structure of the Pt_3_Ni nanoparticles, with an ∼1 nm Pt shell and mixed Pt–Ni alloy core.

Following the characterization at open circuit potential, the catalyst behavior at various potentials was studied by stepping the potential down from 1.28 to 0.1 V_RHE_ (Figure 4). The Pt 4f spectra show a steady decrease in the oxide contributions (Pt^2+^ and Pt^4+^) until ∼0.5 V_RHE_, in line with the reduction currents observed in the CV in Figure 1b. The remaining Pt^δ+^ contribution was previously also observed for pure Pt and is attributed to an adsorbate-induced peak shift of the surface atoms.^32^

**Figure 4:**
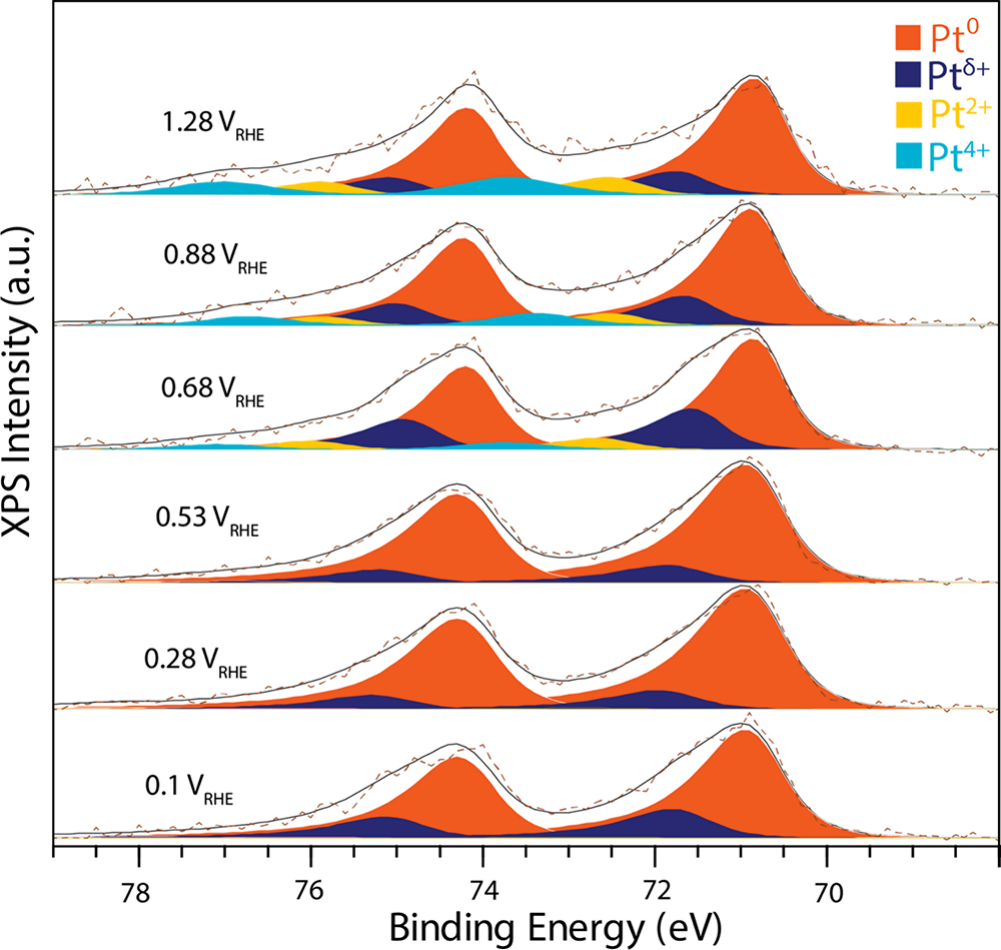
Pt 4f spectra showing the oxidation behavior of Pt in Pt_3_Ni samples as a function of potential while moving from 1.28 to 0.1 V_RHE_, measured with a photon energy of 600 eV.

To track the chemistry of the Ni atoms, we recorded Ni L-edge XAS spectra within the same experiment, i.e., during the potential sequence from 1.28 V_RHE_ to 0.1 V_RHE_. The intensity and multiplet peak pattern of the Ni L-edge are highly sensitive to the charge density of the Ni atoms, enabling the detection of even subtle changes in their electronic structure. To analyze these features, Figure 5a displays normalized Ni L_3_-edge spectra as a function of potential (normalization procedure in the SI Section 10). Over the whole potential range, the spectra show a single asymmetric peak, which is typical for Ni in metal alloys.^47,48^ Importantly, no extra peaks from multiplet features^49^ of Ni^2+^ or Ni^3+^ oxides are observed, showing that even when the Pt shell is oxidized, all Ni remains metallic in the core. This metallic state of the Ni is also confirmed by Ni 2p spectra (Figure S7 in SI Section 12).

**Figure 5:**
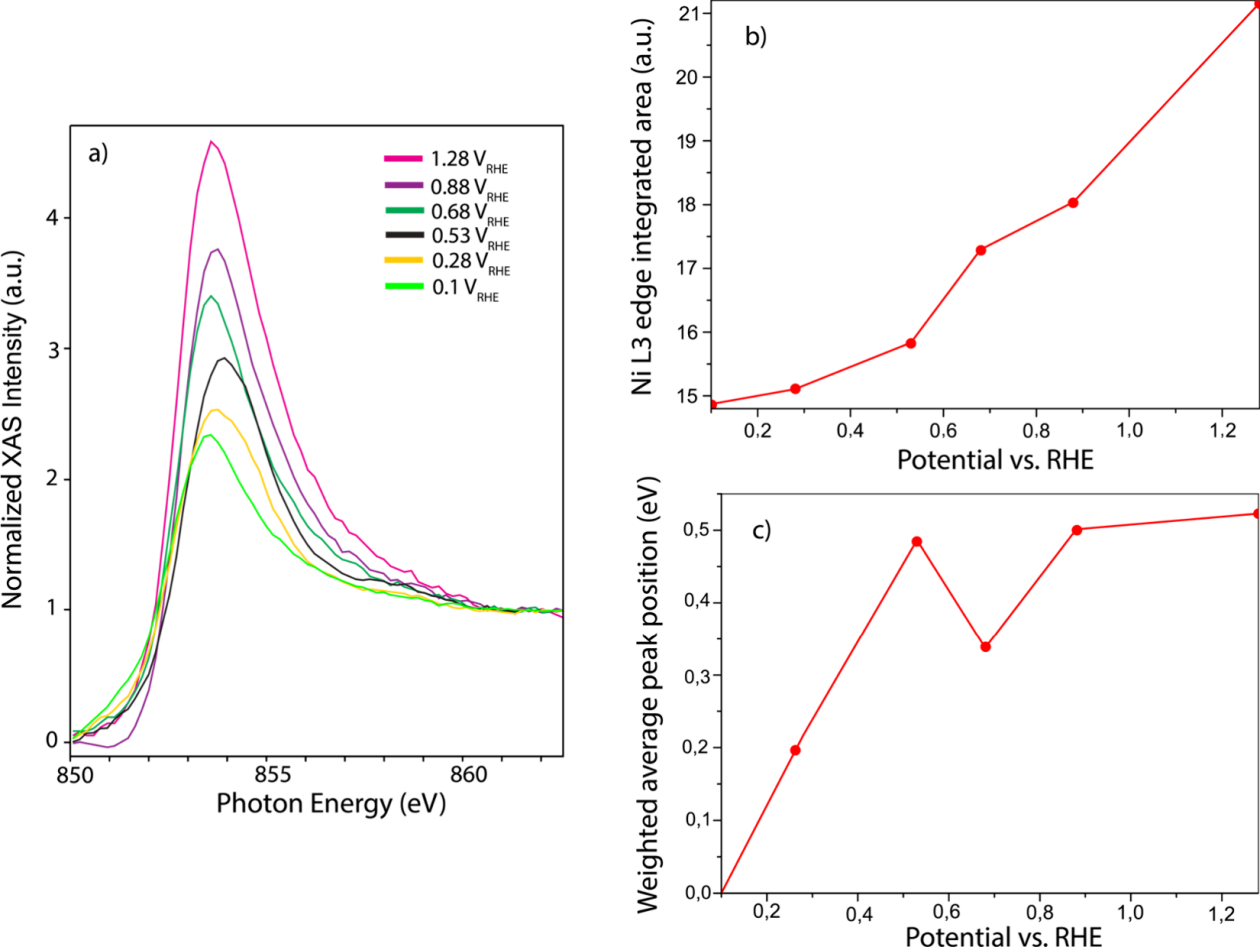
(a) Ni L_3_-edge spectra as a function of potential while moving from 1.28 to 0.1 V_RHE_. (b) Ni L_3_-edge integrated area plotted as a function of potential. (c) Ni L_3_-edge weighted shift in peak position plotted as a function of potential.

However, the Ni L-edge spectra do show a significant decrease in peak intensity as the potential is stepped down from 1.28 to 0.1 V_RHE_, indicating that on the more subtle level, the chemistry of the Ni atoms does change with the applied potential. Since the peak intensity in the Ni L-edge spectrum directly scales with the number of unoccupied Ni 3d states,^47,50–52^ the decreasing intensity indicates that the Ni d-band becomes more electron-rich as the potential is decreased. This can be rationalized based on the surface state of the particles: at low potential (<0.3 V_RHE_), hydrogen atoms are adsorbed on the Pt shell, which do not draw much electron density from the nanoparticles. At intermediate potentials (0.3–0.6 V_RHE_), sulfonate anions from the Nafion membrane and water molecules adsorb,^53–56^ which draw away slightly more electron density. Since the Ni atoms are less noble than the Pt atoms, this electron density is supplied by the Ni atoms, giving them a δ^+^ state. Accordingly, a higher intensity is observed in the Ni L-edge. Above 0.6 V_RHE_, the Pt shell is oxidized, drawing more electron density from the Ni atoms, further increasing their δ^+^ character. Thus, although the Ni atoms are located in the core of the particle, they do play a role in the surface chemistry of the particles, in line with the observed Ni-induced effects in the CV (Figure 1b).

Figure 5a,c shows that the peak position of the Ni L-edge is also affected by the potential-dependent chemical state of the Ni atoms. This can in part be explained by the partial oxidation of the Ni atoms to a δ^+^ state: an increase in Ni 3d vacancies reduces the electron–electron repulsion experienced by the Ni 2p core electrons, leading to a shift in the weighted average peak position of Ni L_3_-edge to a higher energy, as shown in Figure[Fig fig5]c (details of calculation of the weighted average are presented in the SI Section 10). However, a comparison of Figure 5b and c shows that there is a nonmonotonous shift in the Ni L-edge peak position as well as a gradual broadening of the peak, which does not follow a consistent trend as the Ni L-edge intensity. Keeping in mind that the Ni L-edge peak position not only depends on the oxidation state but also on the coordination environment of the Ni atoms,^57^ this suggests that not only the charge density on the Ni atoms but also their coordination environment changes.

To further investigate this potential-dependent restructuring of the particles, we studied the Ni 2p/Pt 4f peak area ratio (Figure 6, details of peak integration and ratio calculations in SI Section 8). This ratio provides an indication of the location of the Ni atoms: a low ratio indicates that the Ni atoms are located deeply in the core. Under these conditions, the Ni 2p signal is strongly attenuated due to scattering of the photoelectrons in the Pt shell, whereas the Pt 4f electrons in the shell can reach the electron analyzer essentially unhindered. If the Ni moves closer to the particle surface, then this attenuation effect in the Ni 2p signal is decreased, resulting in a higher Ni 2p/Pt 4f ratio. This process is clearly observed in Figure 6, which shows that the Ni 2p*/*Pt 4f ratio markedly increases with increasing potential. This means that as the adsorbates on the Pt_3_Ni particles become increasingly electron-withdrawing at higher potentials, the Ni atoms are not only polarized to a δ^+^ charge state but also drawn closer to the surface. Note, however, that even at the highest potentials, the Ni does not become part of the surface, as evidenced by the fact that the Ni atoms are not oxidized to Ni^2+^ at potentials where the Pt oxidizes. Hence, migration of the Ni atoms is a subsurface event, as schematically depicted in Figure 6b. Importantly, the Ni migration is a reversible effect: it can be observed both when the potential is first held at 1.28 V_RHE_ and then stepped down to 0.1 V_RHE_ (red line in Figure 6a) and when the potential is held at 0.1 V_RHE_ and then stepped up to 1.28 V_RHE_ (black line in Figure 6a). Note that the offset between the two lines results from the fact that they were recorded using two different samples with slight variation in catalyst structure.

**Figure 6:**
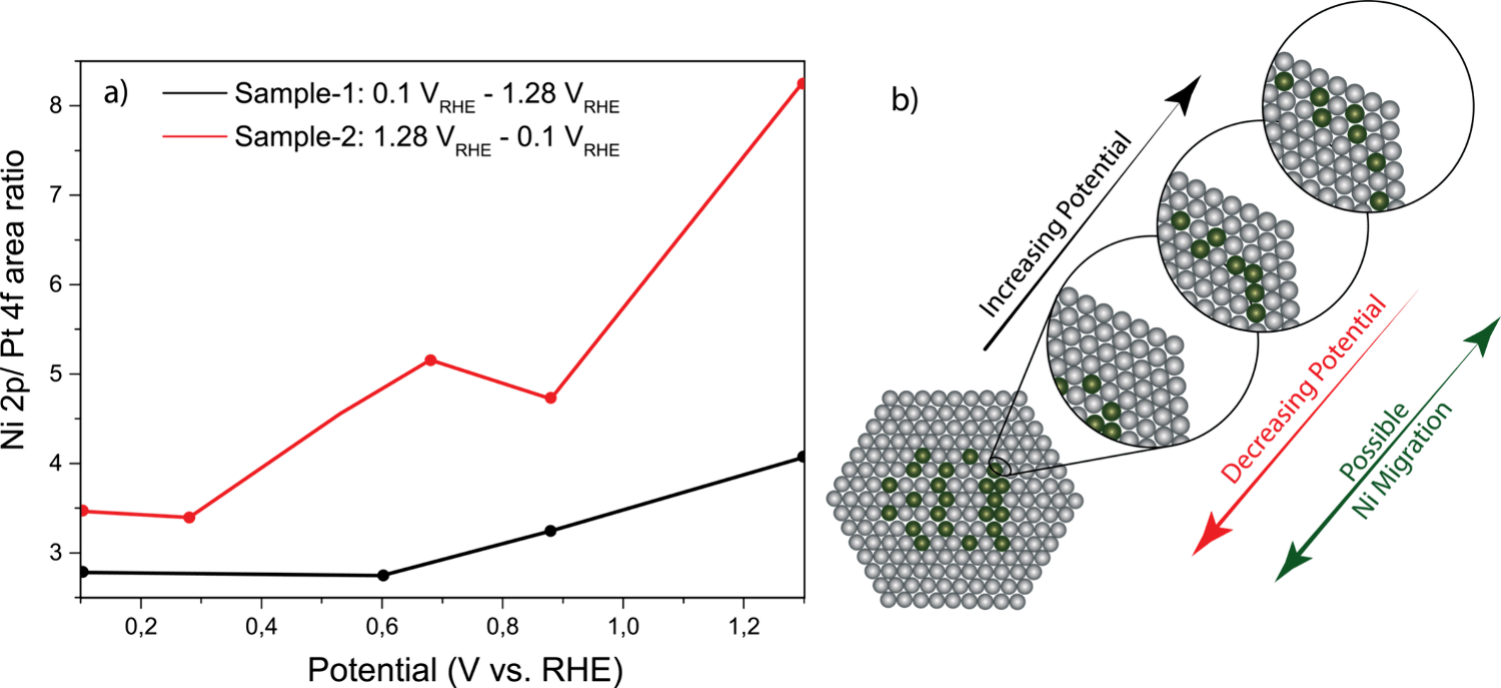
(a) Ni location tracking via the Pt 4f/Ni 2p ratio as a function of potential. (b) Pt_3_Ni nanoparticle structure showing the migration of Ni toward and away from the surface as a function of potential.

The potential-induced migration of Ni causes the thickness of the Pt shell to vary with the applied potential. As discussed in the Introduction, the Pt shell thickness has an important effect on the catalytic properties of Pt alloys.^26,27,58^ In order to probe this effect for our Pt_3_Ni particles, we designed a potential step experiment where the potential was first held at either 0.3 V_RHE_ (thick Pt shell) or 0.9 V_RHE_ (thin Pt shell), followed by a switch to −0.01 V_RHE_ to measure the hydrogen evolution reaction (HER) activity of the particles. As shown in Figure[Fig fig7], there is a marked difference between the two sequences: the HER activity following a hold at 0.9 V_RHE_ is 31% higher than that after a hold at 0.3 V_RHE_. Such a difference is not observed for pure Pt particles, confirming that the effect is caused by Ni migration. The important conclusion that can be drawn from these experiments is that the catalytic properties of Pt_3_Ni electrocatalysts depend on the potentials at which they are used.

**Figure 7:**
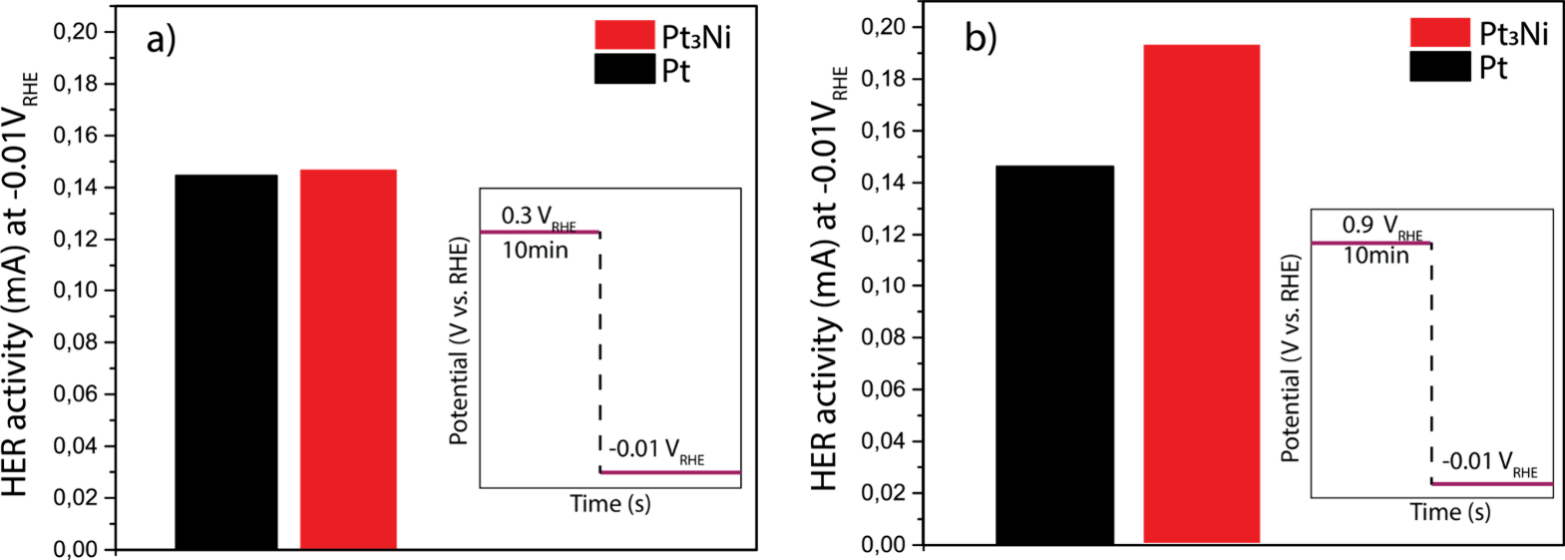
Effect of Ni migration on the hydrogen evolution reaction (HER) activity of Pt_3_Ni. (a) Low potential conditioning at 0.3 V_RHE_ (thick Pt shell) and (b) high potential conditioning at 0.9 V_RHE_ (thin Pt shell). Pure Pt particles are shown for reference. The experiments were conducted in a glass cell in Ar-saturated 0.1 M HClO_4_ with sputter-coated Pt_3_Ni and Pt glassy carbon substrates. The HER current was averaged for 30 s after stabilization of the current to avoid (pseudo)­capacitive contributions.

To obtain insight into the time scale of the Ni migration, the potential step experiment was repeated with different conditioning times at 0.9 V_RHE_. As can be seen in Figure 8, the enhancement of the HER current stabilizes at a conditioning time of about 10 min. This indicates that the Ni atoms can move over several atomic spaces in a matter of minutes to find their equilibrium configuration at 0.9 V_RHE_. Considering that the experiments were carried out at room temperature, this is a remarkable rate. It may therefore be hypothesized that Ni diffusion is accelerated by the adsorption-induced charge transfer from the Ni to the adsorbates, analogous to the Cabrera–Mott mechanism for metal oxide formation.^59^

**Figure 8:**
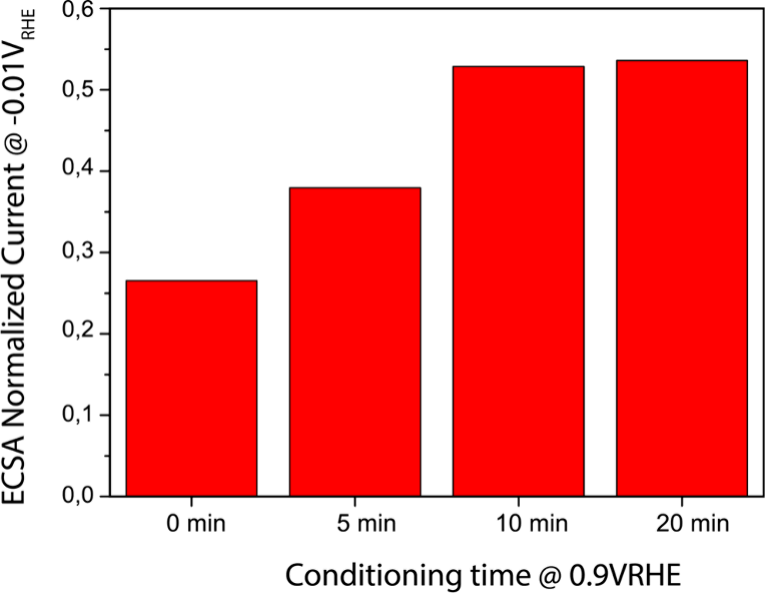
Effect of high potential (0.9 VRHE) conditioning time on the HER activity. The HER current here was averaged over 30 s and has been normalized to the case without the high potential conditioning step, to highlight the relative effect of high potential conditioning on Ni migration and the time scale on which it occurs.

## Conclusions

In conclusion, we have shown that the structure and catalytic properties of Pt_3_Ni nanoparticles are dependent on the applied potential. The restructuring of the particles is subtle: a PtNi_*x*_ core–Pt shell structure is maintained over the entire potential range, but the thickness of the Pt shell varies due to Ni migration. Nonetheless, this restructuring has a marked effect on the catalytic properties of the particles, as probed through the hydrogen evolution reaction. The driving force for Ni migration is the interaction between the Ni atoms in the core and the adsorbates on the particle surface, which involves charge transfer from Ni to the surface. This interaction is strong enough to facilitate Ni migration on a time scale of minutes. Importantly, the adsorbate-interaction-driven alloy restructuring uncovered here is a very general mechanism that one may anticipate to occur for other alloys as well. Therefore, we expect that in situ restructuring of alloys is an important factor to consider in the design of bimetallic electrocatalysts with optimal binding properties.

## Supplementary Material



## Notes

Experimental procedures, data treatment procedures, spectro-electrochemical cell design, electron microscopy analysis, and in situ Ni 2p data (PDF)
