# Supplementary material for: The Potential-Dependent Structure of Pt3Ni Alloy Electrocatalysts and Its Effect on Electrocatalytic Activity
Source: ACS Catal. 2025 Jul 16;15(15):12994–3002. doi: 10.1021/acscatal.5c02601 (PMC12322911; doi:10.1021/acscatal.5c02601)
Supplement: Supplementary file 1 [file cs5c02601_si_001.pdf]

The potential-dependent structure of Pt_3Ni alloy electrocatalysts and its effect on electrocatalytic activity

Hassan Javed[1*], Kees Kolmeijer[1], Nipon Deka[1], Matthijs A. van Spronsen[2], Marijn A. van Huis[3], Athira Lekshmi Mohandas Sandhya[4], Ivan Khalakhan[4], Rik V. Mom[1*]

[1]Leiden Institute of Chemistry, Leiden University, PO Box 9502, 2300 RA Leiden, The Netherlands

[2] Diamond Light Source Ltd., Harwell Science and Innovation Campus, Chilton, Didcot, OX11 0DE, UK

[3]Debye Institute for Nanomaterials Science, Utrecht University, Princetonplein 5, 3584 CC Utrecht, The Netherlands

[4]Department of Surface and Plasma Science, Faculty of Mathematics and Physics, Charles University, V Holešovičkách 2, 180 00 Prague 8, Czech Republic

*h.j.nagra@lic.leidenuniv.nl

*r.v.mom@lic.leidenuniv.nl

Supplementary Information

1. Nafion Membrane Activation

The Polymer electrolyte membrane with sputtered Pt nanoparticles is Nafion 117 supplied by Sigma-Aldrich. The membrane was cut into 1 cm squares, followed by the process of activation and cleaning. The Nafion membrane needs to be cleaned of the carbonaceous impurities. For this purpose, the samples were treated with a 3% H_2O_2 solution at 80^oC for 2 hr and then with dilute H_2SO_4 solution (0.5 M) for the same duration and at the same temperature. Following the cleaning procedure, the membranes were rinsed with deionized water and stored dry.

2. Glassy Carbon (GC) electrode preparation

5 mm diameter GC discs were polished with polycrystalline diamond suspension (MetaDi^{TM} Supreme, Buehler) to a mirror finish. The electrodes were then ultrasonicated in deionized water to remove polishing solution residue. Clean GC discs were dried in an Argon gas stream. Pt and Pt_3Ni nanoparticles were sputter deposited onto the cleaned discs.

3. Membrane Electrode Assembly Preparation

Pt_3Ni alloy and Pt metal were used to prepare the membrane electrode assembly (MEA) by sputter depositing the catalyst nanoparticles onto activated Nafion 117 polymer exchange membranes.

For Pt MEA samples, a Pt target (ChemPUR GmbH, 99.5% purity) and a DC magnetron sputter coater 208HR by Cressington (Watford, UK), were employed. A sputtering current of 40 mA was used with the sputter chamber pressure maintained at ~$4x10^{-4}$ Pa. The sputtering process lasted for 2 minutes and 45 seconds to deposit a 5 nm thick layer of Pt.

Pt_3Ni alloys were deposited using two circular TORUS magnetrons (Kurt J. Lesker) operating simultaneously. 2" Pt (99.99% Safina) and 2" Ni (99.99% Kurt J. Lesker) targets were employed for the sputtering of Pt and Ni, respectively. Prior to deposition, the sputtering chamber was evacuated to a base pressure of 1×10^{-4} Pa. Sputtering was then performed in a 2.6 Pa argon atmosphere in DC mode, with the power set to 28 W for Pt and 30 W for Ni to achieve the desired alloy composition. The deposition lasted for 1 minute and 35 seconds to attain a 5 nm thickness of the deposited layer.

4. Graphene deposition on the Nafion MEA

Following the preparation of the MEA, a graphene bi-layer is deposited on top of the Pt nanoparticles to impede the escape of the electrolyte during the spectroscopic measurements in the XPS vacuum chamber. This graphene serves as both an X-ray/photoelectron transparent window (>300 eV) and as the electrical contact with the Pt nanoparticles. Graphene is deposited using a wet chemical method. For this

purpose, graphene supported on copper (Graphenea SA) was etched in a 40 g/L solution of ammonium persulfate overnight, dissolving copper and leaving the graphene layer floating on the liquid surface. The solution was then exchanged with pure water and the floating graphene was scooped off the surface of the water with the MEA. The prepared sample with graphene was dried at room temperature and proper placement of graphene was ensured on the membrane by visual inspection.

5. EDX Sample Analysis

The sample composition was verified through bulk-sensitive Energy-Dispersive X-ray Spectroscopy (EDX), utilizing an XFlash detector (Bruker) integrated into the Mira 3 Scanning electron microscope (Tescan) operated at electron energy of 30 keV.

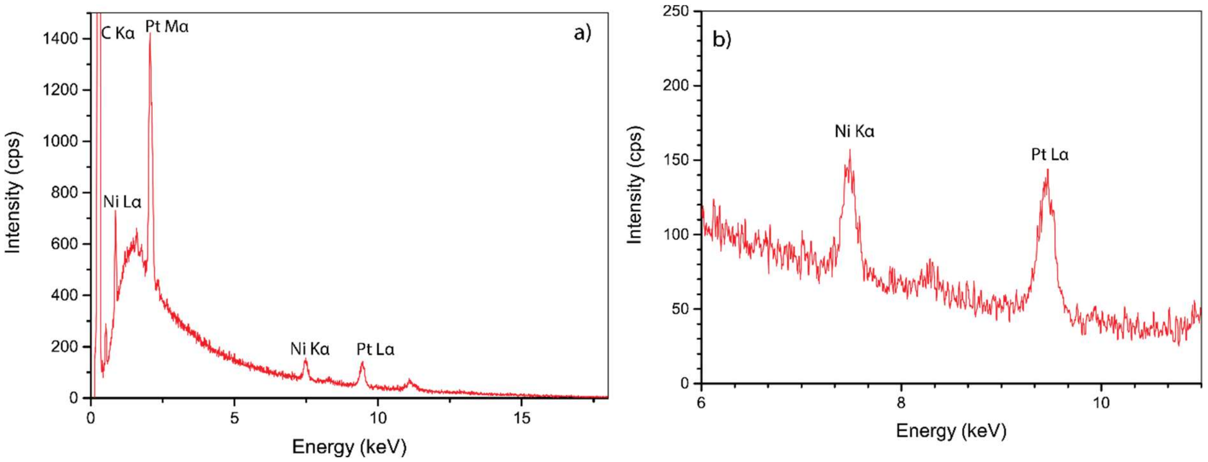

Figure S1: Energy Dispersive X-ray (EDX) analysis of as-prepared Pt_3Ni samples

The bulk composition of the as-deposited Pt_3Ni layer was quantified from the EDX spectra shown in Figure S1 by integrating the Ni $K\alpha_1$ (7.480 keV) and Pt $L\alpha_1$ (9,442 keV) lines. The calculated composition was $Pt_{73}Ni_{27}$, close to the intended Pt_3Ni composition.

6. Cell Design

All experiments were performed in the newly designed Spectro-electrochemical flow-cell shown in Figure S2. A titanium top plate secures the MEA in the cell. A Pt wire is used as a counter electrode and Ag/AgCl electrode is used as a reference. Electrolyte connections are present at the rear of the cell, to which the electrolyte lines are connected via PTFE fittings. The main body of the cell is made out of PEEK. The cross section of the cell shown on the right hand side in Figure S2 shows the internal electrolyte cavity and the circulation. A minimum dead volume is maintained inside the cavity, minimizing the distance between the electrodes and the cell resistance.

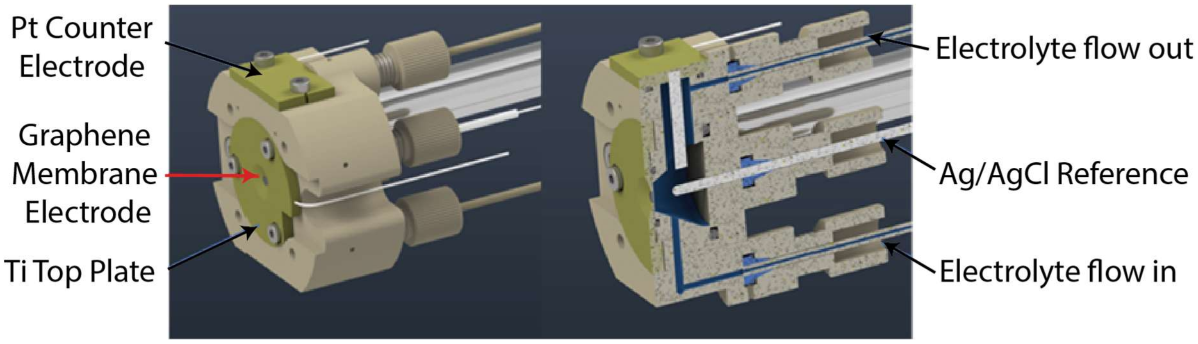

Figure S2: Schematic of the three-electrode spectro-electrochemical cell set-up for in-situ X-ray spectroscopy.

7. Pt 4 f XPS Data fitting

The raw XPS data was processed using CasaXPS Version 2.3.23. A Tougaard background subtraction was used for all the datasets. To fit the data, we employed a fitting model established in previous work[1,2]. The Lorentzian LF line shape was employed, which is an extension of the Lorentzian LA line shape, the purpose of which is to limit the intensity of the asymmetric tails. It fits the data on the basis of 4 parameters;α, β, w and m. Varying 'α' and 'β' results in increasing or decreasing the spread of the tail for the Lorentzian curve, thereby affecting the steepness of the edges of the line shape. 'm' is the integer specifying the Gaussian convolution with the Lorentzian function and 'w' is the dampening factor to force the tails of to zero towards the limits of integration. An asymmetric form of Lorentzian LF line shape function was used for fitting the Pt 4f spectra. Doublets for Pt^0 $Pt^{\delta+}$, Pt^{2+} and Pt^{4+} were used, with a constant spin-orbit splitting of 3.34 eV and a peak area ratio of 3:4 for the $4f_{5/2}$ and $4f_{7/2}$ peaks. The details of the fitting parameters are shown in the Table S1.

The ranges for the peak positions of different Pt oxidation states were determined in accordance with the literature, as summarized by Saveleva *et al.*[3], and previous work[1]. In order to accommodate the variations in the configuration of the beam energy for different experiments, a slight variation in the binding energy for Pt^0 (± 0.1 eV) was permitted. The lineshape and FWHM for the Pt^0 and $Pt^{\delta+}$ contributions was determined at the lowest potential, where only Pt^0 and $Pt^{\delta+}$ are present (Pt^0 in the core, $Pt^{\delta+}$ on the surface due to contact with adsorbates (e.g. H_2O, OH, O, R-SO_3^-) . Note that the line shape is asymmetric, as generally observed for metallic platinum, due to electron-hole pair excitations.

For Pt^{2+} the same line shape as for Pt^0 was assumed. The Pt^{4+} line shape, peak position, and FWHM were determined by free fitting of a highly oxidized 5nm Pt sample produced at 1.7 V_{RHE}, which displays a well-resolved Pt^{4+} peak. The consistency of the fit model was ensured by applying it to several data sets.

	Pt^0	$Pt^{\delta+}$	Pt^{2+}	Pt^{4+}
**Line shape**	LF(0.9,2,60,50)	LF(0.9,2,60,50)	LF(0.9,2,60,50)	LF(3,3,30,10)
Peak Position	71.2 - 70.9	72 - 71.2	72.7 - 72	Determined at highest potential of the dataset
FWHM	Free	Same as Pt^0	Same as Pt^0	Determined at highest potential of the dataset

Table S1 : Fitting parameters for Pt 4f spectral decomposition

8. Pt 4*f* / Ni 2*p* peak area integration

During our experimental work at Diamond light source B-07 beamline, an exit slit gap of 0.025 mm (grating = 600 lines/mm) was used. The electron analyzer (SPECS PHOIBOS NAP 150) coupled to the end station uses 3.0 x 25 mm entry and 4:3 x 25 mm (open) exit slit. Pt 4f spectra were recorded at 600 eV excitation energy with 10 eV energy while the Ni 2p spectra were measured at 1900 eV excitation energy with 50 eV pass energy at same measurement spot. The pass energy and exit slit settings are not the lowest possible but represent typical values used to achieve high resolution with reasonable intensity. A different measurement spot was chosen for every potential to avoid beam damage to the Nafion membrane. A Tougaard background subtraction was applied to the spectra and the area under the curve was integrated in CasaXPS. The Ni 2p / Pt 4f ratio was calculated by simply dividing the area under the Pt 4f and Ni 2p spectra.

9. Pt_3Ni electron microscopy Images

Error! Reference source not found. shows the SEM images taken for an 'as prepared' Pt_3Ni MEA sample. A clear contrast between the graphene covered (dark) and bare catalyst surface (bright) on the MEA can be seen in **Error! Reference source not found.**a by imaging at the edge of the deposited graphene. An area in the middle the graphene window is shown in Figure 3b. The lack of microscopic tears in the graphene confirms the quality of the window. **Error! Reference source not found.**d shows a higher magnification. Here, the contrast originates from the catalyst layer (light) with respect to the non-conductive underlying Nafion (dark), both of which are imaged through the (fairly) electron-transparent graphene window. A clear cracking pattern can be observed, which is formed when the Pt_3Ni-coated Nafion membrane is inserted in water to pick up the graphene window. Nafion swells by about 10% in water, cracking up the film.

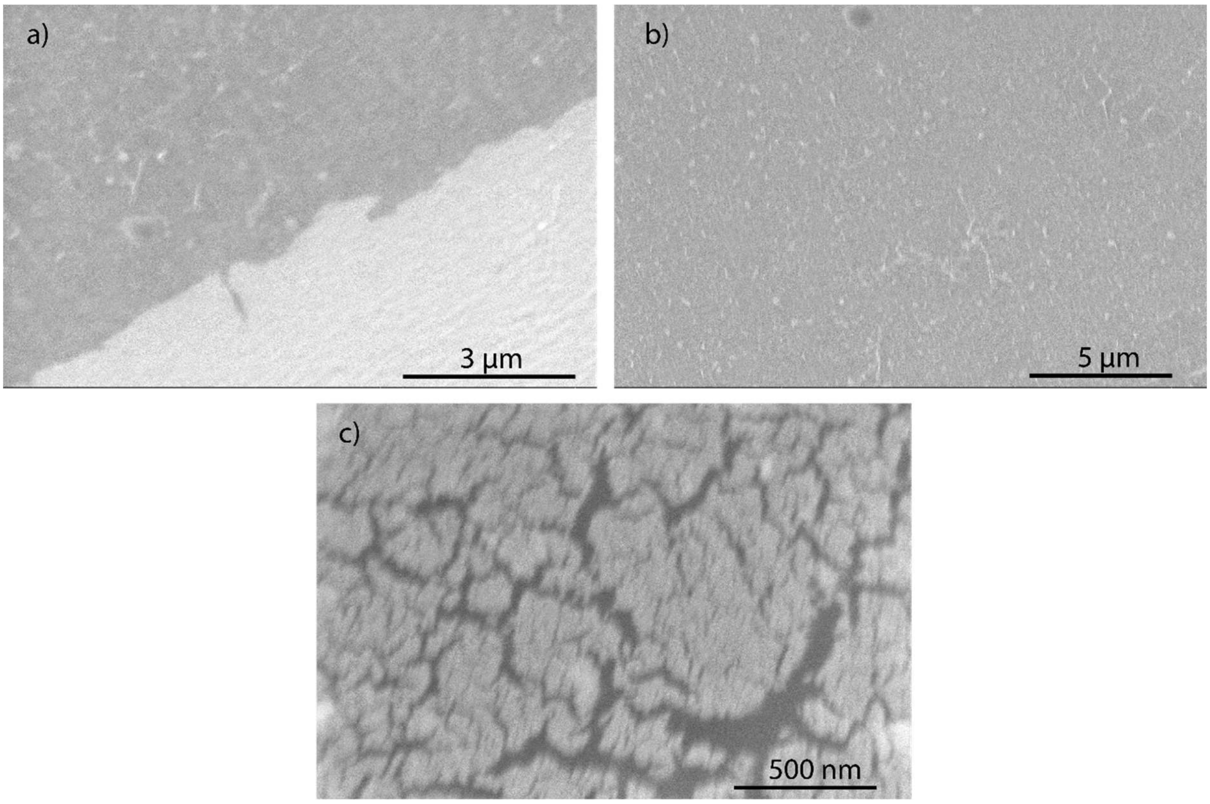

Figure S3: SEM Images for Pt_3Ni catalyst sputter deposited onto cleaned and activated Nafion membrane

Unfortunately, attaining higher resolution images of the MEA was not possible due to charging and beam damage of the Nafion membrane. However, Figure S4 shows a collection of SEM images for a similar catalyst loading deposited on a polished glassy carbon surface.

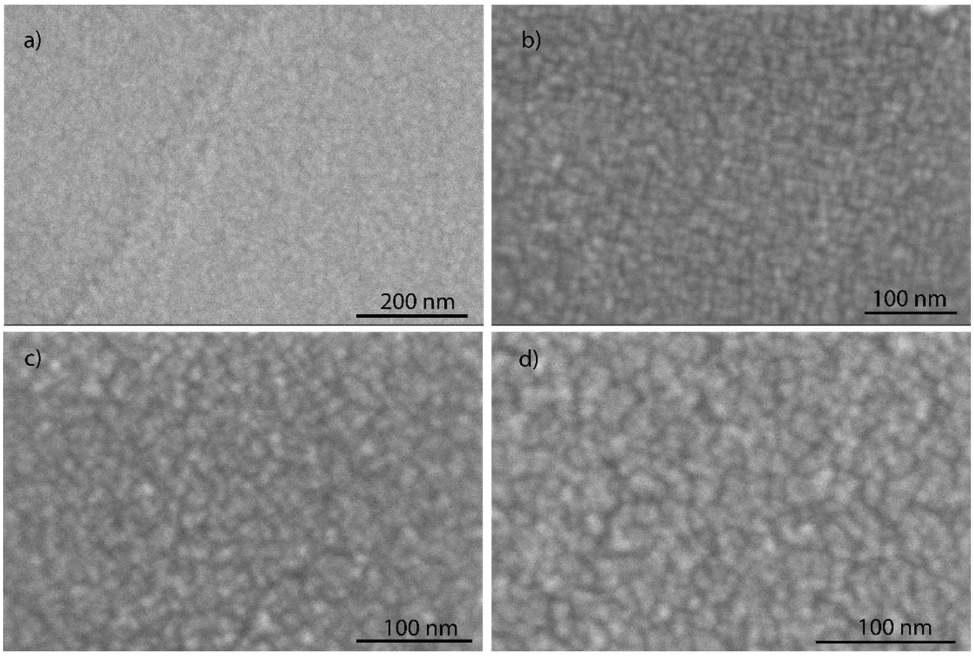

Figure S4: SEM Images for Pt_3Ni catalyst sputter deposited onto polished glassy carbon disk

For the TEM measurements, copper TEM grids (TED PELLA Inc. GSB50 Grids, coated with holey carbon) were covered with a thin Nafion film by dip coating in a resin solution (5 wt% Nafion in lower alcohols, Sigma Aldrich), which was diluted 20x in ethanol. The Pt_3Ni electrocatalyst was sputter deposited onto the Nafion coated TEM grids following the same procedure as described before. Figure S5 a-c show the high resolution TEM images, where it can be clearly seen that Pt-Ni catalyst particles have an average size of 3-4 nm.

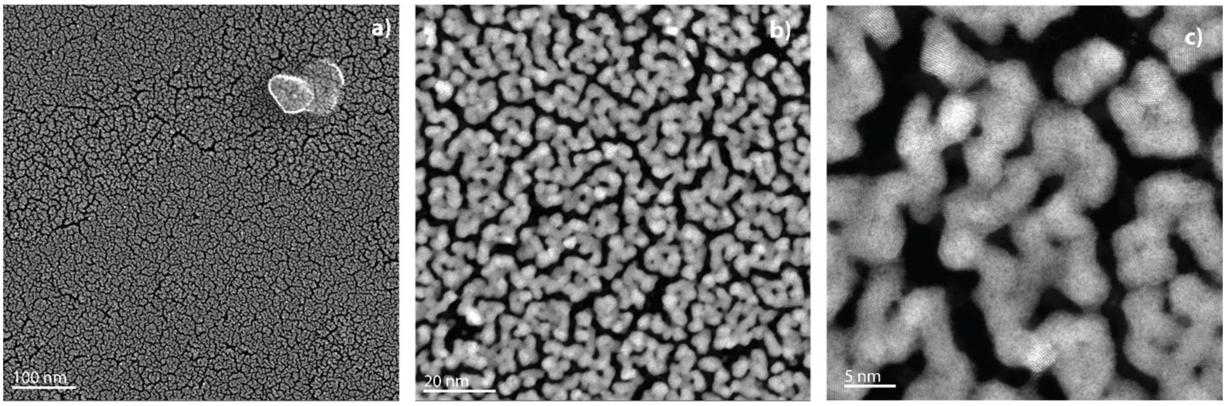

Figure S5: High-resolution TEM analysis of the sputtered Pt3Ni particles in pristine state. The average particle size is in the range of 3-4 nm.

10. XAS Data processing and arctan correction for Ni L_3-edge

Ni L-edge XAS data was processed using Athena (version 0.9.26). First, a linear pre- and post-edge correction was applied to remove the background. Subsequently, the spectrum was normalized to 1 at the L_3 edge jump. Due to the spin orbit splitting ratio of 2:1 between the $2p_{3/2}$ and $2p_{1/2}$ core levels that are excited in the L_3 and L_2 edges, respectively, the edge jump of the L_2 edge should be 0.5 when the L_3 edge is normalized to 1 (note that this only holds for the edge jumps, not the white lines, which may have deviating branching ratios). We used this expected value of 0.5 for the L_2 edge jump to verify the correctness of the processing. In addition, we checked that the L_2-edge white line intensities showed the same trends as the L_3-edge white line intensity.

For the analysis of the intensity and weighted average peak position of the Ni L_3-edge white line (Figure 5 in the main text), we subtracted the edge jump from the normalized Ni L_3-edge spectra, thus isolating the whiteline. An arctan line shape subtraction was used for this purpose, as shown in Figure S6a, red curve. Subtraction resulted in the pure white line spectrum in Figure S6b. A similar background correction was applied to Ni L_3-edge for other potential steps and the corrected curves were integrated to calculate the Ni L_3-edge area for Figure 5b (main text).

To distinguish the minor peak shifts in Ni L_3-edge as a function of potential, a weighted averaged peak position was calculated for each step potential curve (Figure 5c, main text). For this purpose, integrated Ni L_3-edge curves were used as shown in Figure S6c

for 1.28 V_{RHE}. The dotted lines in Figure S6c coincides with the middle (half) of the integrated intensity on the *y*-axis. The corresponding photon energy on the *x*-axis was used as the weighted average peak position in Figure 5c in the main text.

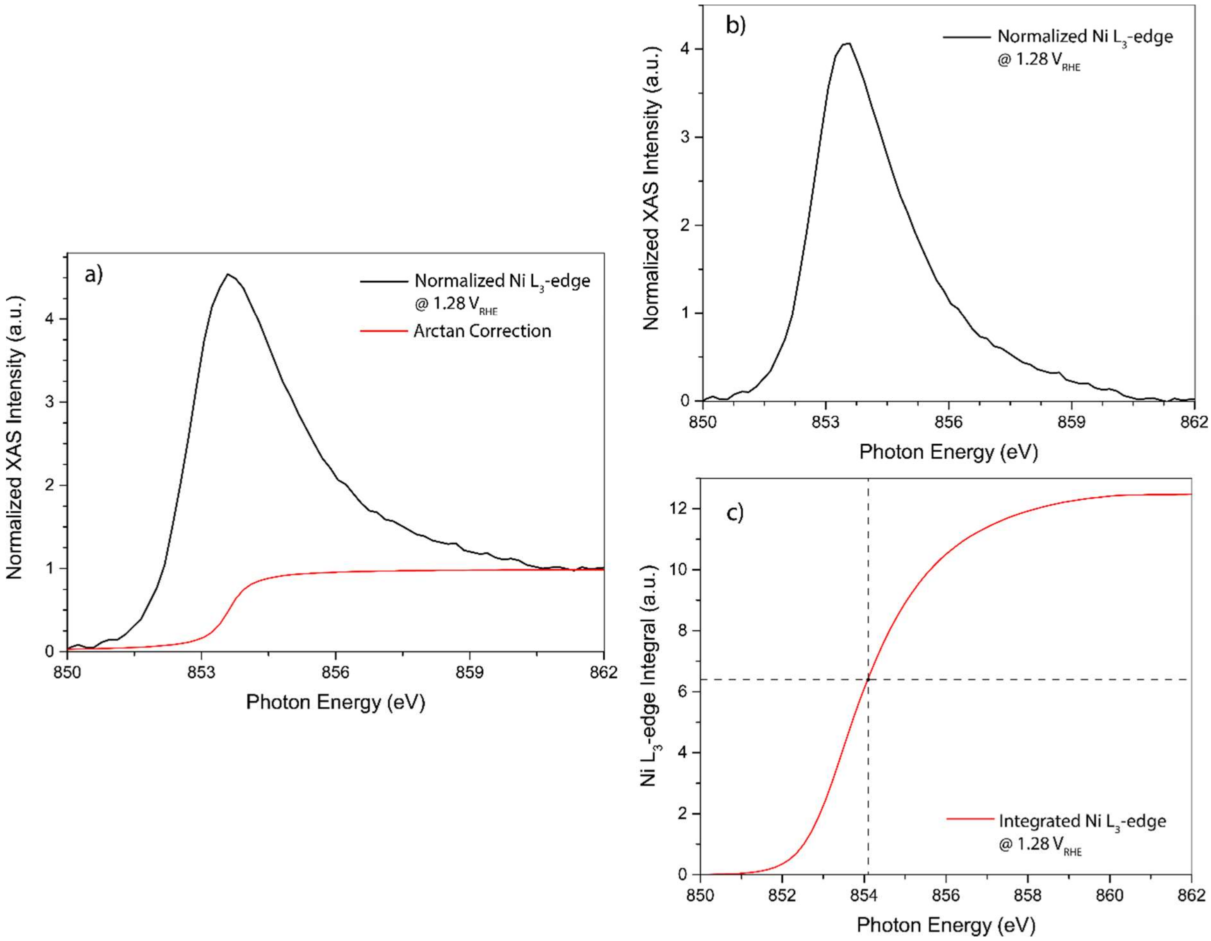

Figure S6: Arctan correction for the analysis of the Ni L_3-edge spectra; a) shows an example spectrum (black curve) and an arctan function to be subtracted (red curve), b) shows the resultant curve after the subtraction and c) shows the integrated version of b), where the dotted line indicated the weighted average peak position on the x-axis

11. Pt & Pt_3Ni Cyclic voltammogram and activity

Pt and Pt_3Ni catalysts were tested in a glass cell for their electrochemical behavior measured in Argon purged 0.1 M H_2SO_4. Figure S7 shows similar voltametric behavior in the glass cell as observed in the spectro-electrochemical experiments discussed in the main text (Figure 1b), where weak adsorbate interaction in Pt_3Ni, as well as higher oxidation current as compared to bulk Pt catalyst, can be observed.

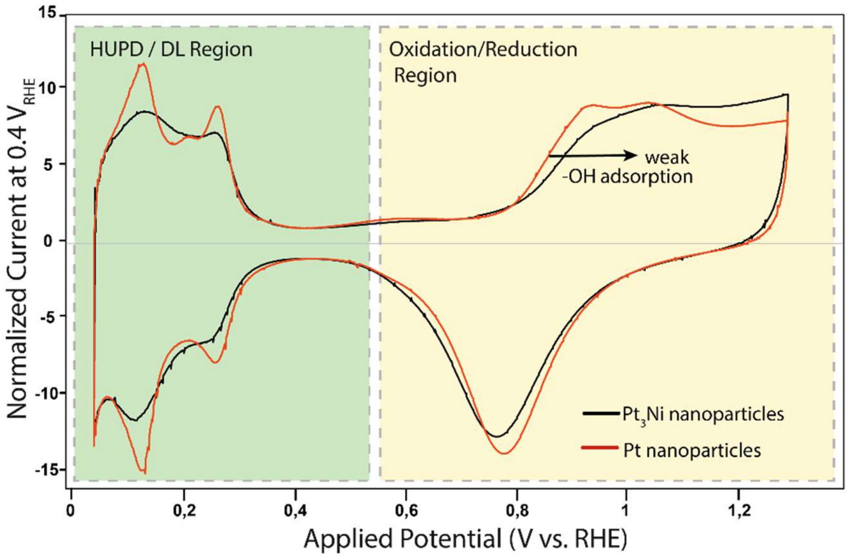

Figure S7: Cyclic voltammograms of Pt and Pt_3Ni catalyst nanoparticles deposited on glassy carbon substrate in Ar purged 0.1M H_2SO_4 highlighting Hydrogen Underpotential Deposition (HUPD), Double Layer (DL) and oxidation/reduction regions

12. Ni 2*p* potential dependent XPS Spectra

Figure S8 shows Ni 2*p* XPS spectra measured at different potentials. The binding energy is consistent with metallic Ni.

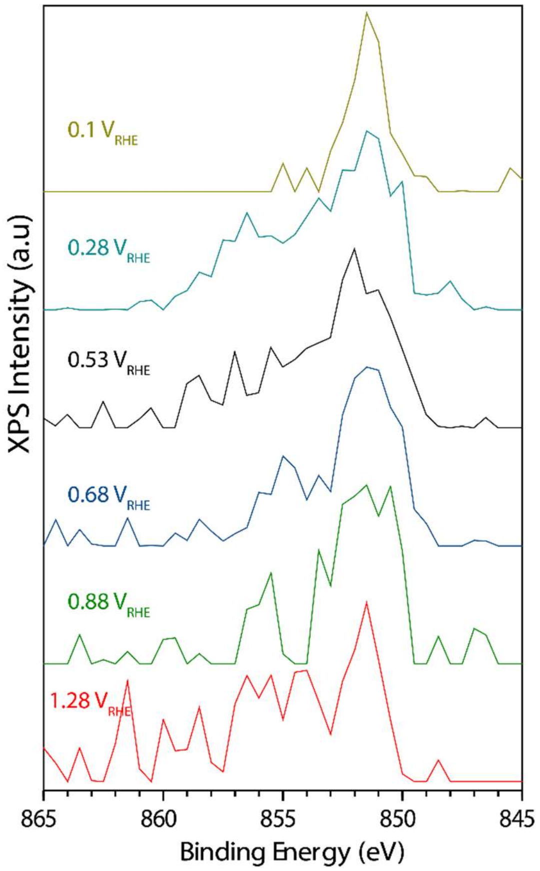

Figure S8: Ni 2p XPS spectra as a function of potential at 1900 eV excitation energy

References

(1) Javed, H.; Knop-Gericke, A.; Mom, R. V. Structural Model for Transient Pt Oxidation during Fuel Cell Start-up Using Electrochemical X-Ray Photoelectron Spectroscopy. *ACS Appl Mater Interfaces* **2022**, *14* (31), 36238–36245. https://doi.org/10.1021/acsami.2c09249.

(2) Mom, R.; Frevel, L.; Velasco-Vélez, J. J.; Plodinec, M.; Knop-Gericke, A.; Schlögl, R. The Oxidation of Platinum under Wet Conditions Observed by Electrochemical X-Ray Photoelectron Spectroscopy. *J Am Chem Soc* **2019**, *141* (16), 6537–6544. https://doi.org/10.1021/jacs.8b12284.

(3) Saveleva, V. A.; Papaefthimiou, V.; Daletou, M. K.; Doh, W. H.; Diebold, M.; Zafeiratos, S.; Savinova, E. R.; Ulhaq-bouillet, C.; Diebold, M.; Zafeiratos, S.; Savinova, E. R. Operando Near Ambient Pressure XPS (NAP-XPS) Study of the Pt Electrochemical Oxidation in H 2 O and H 2 O/O 2 Ambients. *Journal of Physical Chemistry C* **2016**, *120* (1), 15930–15940. https://doi.org/10.1021/acs.jpcc.5b12410.
